# Dissociation of Endogenous Pain Inhibition Due to Conditioned Pain Modulation and Placebo in Male Athletes Versus Nonathletes

**DOI:** 10.3389/fpsyg.2020.553530

**Published:** 2020-09-18

**Authors:** Maria Geisler, Marco Herbsleb, Karl-Jürgen Bär, Thomas Weiss

**Affiliations:** ^1^Department of Clinical Psychology, Friedrich-Schiller-University Jena, Jena, Germany; ^2^Department of Sports Medicine and Health Promotion, Friedrich-Schiller-University Jena, Jena, Germany; ^3^Department of Psychosomatic Medicine, University Hospital Jena, Jena, Germany

**Keywords:** pain modulation, endurance athletes, placebo, conditioned pain modulation, interoceptive awareness

## Abstract

Animals and humans are able to inhibit pain by activating their endogenous pain-inhibition system. Endurance athletes possess a higher pain-tolerance threshold and a greater conditioned pain modulation (CPM) effect than nonathletes, suggesting better endogenous pain inhibition. In addition to CPM, placebo is another prominent paradigm used to test endogenous pain inhibition. However, whether the placebo effect and the CPM effect share the same mechanisms of pain inhibition has not been investigated. If there is a shared mechanism, then endurance athletes should show not only a better CPM effect than nonathletes but also a greater placebo effect. Here, we investigated 16 male endurance athletes and 17 male nonathletes in well-established placebo and CPM paradigms to assess whether endurance athletes have a better endogenous pain-inhibition system than nonathletes. As expected, we find a significantly greater CPM effect in athletes than in nonathletes. In contrast, we could only find a significant placebo effect in nonathletes. Explorative analyses reveal negative associations between the placebo effect and heart rate variability as well as between the placebo effect and interoceptive awareness. Together, the results demonstrate a dissociation of endogenous pain inhibition of CPM and placebo effect between endurance athletes and nonathletes. This suggests that both effects are based, at least in part, on different biological mechanisms.

## Introduction

The human body is able to inhibit pain without pain-relieving medications by activating its own endogenous pain-inhibition system (Basbaum and Fields, 1978). There are several ways to reduce pain without medication, for example, by focal electrical stimulation of the brain (Mayer and Liebeskind, 1974), distraction or disengagement (Valet et al., 2004; Romero et al., 2013), stress (Yilmaz et al., 2010), exercise (Koltyn, 2000), placebo (Amanzio and Benedetti, 1999), or localized painful stimulation [conditioned pain modulation (CPM); Yarnitsky et al., 2010]. Of these, placebo and CPM are probably the most frequently used paradigms to assess the ability of humans’ endogenous pain inhibition (Damien et al., 2018).

There is evidence that the placebo effect varies tremendously among individuals ranging from no change in pain perception (nonresponders) to placebo-induced analgesia (Enck et al., 2013). Among others, the individual placebo effect depends on the opioid-modulated strength of functional coupling between the dorsolateral prefrontal cortex (DLPFC), the rostral anterior cingulate cortex (rACC), and the periaqueductal gray (PAG), a key structure of the descending pain-inhibition system (Bingel et al., 2006; Eippert et al., 2009). In an fMRI study, Sprenger et al. (2011) showed that the CPM effect is also modulated in an opioid-dependent manner by higher-order brain regions. Specifically, they find a strengthened functional coupling between the subgenual anterior cingulate cortex (sACC) and the PAG. Furthermore, the individual strength of the coupling between sACC and PAG predicts the CPM effect. This prediction was abolished when naloxone, an opioid antagonist, was administered. Together, these results suggest that hypoalgesia elicited through the CPM and placebo effects might share the same mechanisms of pain modulation.

There is much evidence that a reduction in endogenous pain inhibition is a risk factor for developing chronic pain (Edwards, 2005). A meta-analysis that includes 30 studies finds a large effect of reduced CPM in chronic pain patients compared to healthy controls (Lewis et al., 2012). Compared to chronic pain patients, who unwillingly suffer from uncontrollable and unpredictable pain, endurance athletes voluntary engage in training sessions and competitions known to elicit pain. These athletes need to modulate arising pain to obtain rewards, such as personal bests and competition wins (Flood et al., 2017). Indeed, in a meta-analysis, Tesarz et al. (2012) found that endurance athletes have higher pain-tolerance thresholds than nonathletes. Furthermore, recent studies show that endurance athletes have an enhanced CPM effect compared to healthy nonathletes (Geva and Defrin, 2013; Flood et al., 2016; Geva et al., 2017). Together, a higher pain-tolerance threshold and a greater CPM effect in endurance athletes suggest stronger endogenous pain-inhibition ability compared to healthy nonathletes. If this assumption of a better endogenous pain-inhibition system in endurance athletes is correct and if the placebo and CPM effects are based on the same mechanisms of pain modulation (Sprenger et al., 2011; Damien et al., 2018), then endurance athletes should also show a greater placebo effect than nonathletes.

In the present study, we investigate whether endurance athletes have a better endogenous pain-inhibition system than nonathletes by conducting a CPM and placebo paradigm in healthy endurance athletes and nonathletes. To depict the entire range of painful stimulation (from pain threshold to pain-tolerance threshold), we applied a pressure pain stimulus-response curve as the test stimulus (TS) during the CPM paradigm. We used the serial gatekeeping procedure (Turk et al., 2008) to test our hypotheses in the following hierarchical order: we hypothesized (H1) that athletes perceive pressure pain as less painful than nonathletes and that this difference is more pronounced the higher the stimulation intensity is. Thus, athletes should have a right-shifted pressure pain stimulus-response curve and a lower slope of stimulus response curve. As this hypothesis refers to the results of a meta-analysis (Tesarz et al., 2012) and therewith is based on the most convincing evidence (comparing all four hypotheses), an acceptance of this hypothesis (by rejecting the H0 that there are no differences between groups) should be most likely. When the H1 can be accepted, we can assume the success of our study manipulation and continue with testing (H2) that a greater CPM effect occurs in athletes compared to nonathletes. The main purpose of our H2 is to replicate a handful of recent studies that demonstrate endurance athletes have an enhanced CPM effect compared to healthy nonathletes (Geva and Defrin, 2013; Flood et al., 2016; Geva et al., 2017). Only when this hypothesis is accepted (by rejecting the H0 that there are no differences between groups), can we assume that endurance athletes have a better endogenous pain-inhibition system and continue with testing (H3) that there is a greater placebo effect in athletes compared to nonathletes. H3 has not yet been tested. It is based on the assumption that endurance athletes have a better endogenous pain-inhibition system than nonathletes (tested by H1 and H2) and that the CPM effect and the placebo effect are based on the same mechanisms of pain modulation (Sprenger et al., 2011; Damien et al., 2018). When H3 is accepted (by rejecting the H0 that there are no differences between groups), we can test our last hypothesis (H4) that a negative association exists between the CPM effect (by definition, a negative value representing CPM-induced hypoalgesia) and the placebo effect (by definition, a positive value representing placebo-induced hypoalgesia). An acceptance of H4 would support the suggestion that the CPM and placebo effects are related but would not necessarily conclude that both effects share a physiological mechanism.

## Materials and Methods

### Participants

**Figure 1:**
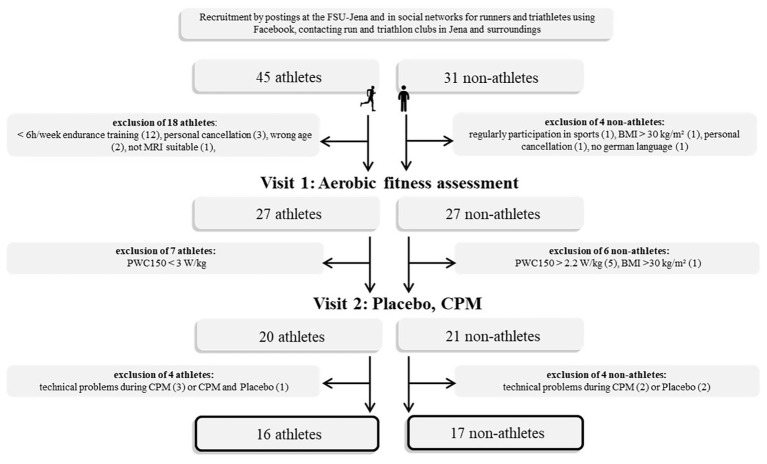
Flow chart of data acquisition. FSU, Friedrich-Schiller-University; PWC, physical work capacity; CPM, conditioned pain modulation; BMI, body mass index.

Participants were recruited by advertisements posted at the University of Jena, by social networks for runners and triathletes on Facebook, and by directly contacting running and triathlon clubs in and nearby Jena. Only male athletes (long-distance runners and triathletes) are included in the study to prevent menstrual-related influences to pain processing (Riley et al., 1999), athletic performance (Lebrun et al., 1995), and mood (Collins et al., 1985). Inclusion criteria for athletes were as follows: age 18–40 years, body mass index (BMI) 18.5–30 kg/m^2^, no pain disorder, psychiatric, or neurological disease. For athletes, we required at least 6 h/week endurance training for the last 3 years with no sign of exercise-dependence risk [total score on the German version of the exercise dependence scale (EDS-G) less than 78; Müller et al., 2013] and physical work capacity during heart rate of 150 bpm (PWC150) ≥ 3.0 W/kg. The inclusion criteria for nonathletes were no regular participation in any kind of sports and PWC150 ≤ 2.2 W/kg. The final sample size included 16 male athletes (age: 27.9 ± 5.0 years, BMI: 22.9 ± 1.6 kg/m^2^) and 17 nonathletes (age: 26.9 ± 6.3 years, BMI: 24.1 ± 3.2 kg/m^2^), who did not differ significantly in mean age and mean BMI. The sample size is in line with previous research reporting significant differences of the CPM effect between athletes and nonathletes (Geva and Defrin, 2013; Flood et al., 2016). Detailed comparisons of athletes’ and nonathletes’ demographic characteristics are given in Supplementary Table S1 and Figure 1. Subjects were paid for participation (25 €). The ethics committee of the Faculty of Social and Behavioural Sciences of the Friedrich Schiller University Jena approved the study. The study was performed in accordance with the Helsinki guidelines. All subjects signed informed consent.

### Study Design

**Figure 2:**
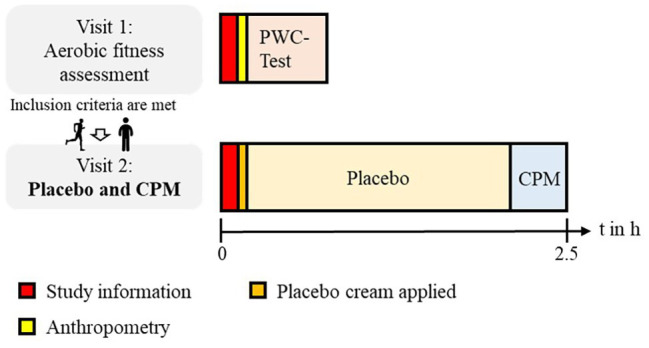
Study design. Using a within-subject design, each participant was investigated on 2 separate days. On visit 1, a PWC test was conducted, and on visit 2 the main experiment (placebo paradigm and CPM paradigm) took place.

Using a within-subject design, each participant was investigated in 2 separate days. On visit 1, a physical work capacity (PWC) test was conducted, and on visit 2, the main experiment (placebo and CPM paradigms) took place. In between both examination days, participants filled out questionnaires assessing psychological variables. On day 2, all subjects underwent a standard placebo paradigm (Wager et al., 2004; Eippert et al., 2009; Stein et al., 2012) and, subsequently, a classical CPM paradigm. The mean time delay between both days was 22 ± 37 days (see Figure 2).

#### Aerobic Fitness Assessment

On visit 1, subjects were informed of the study’s procedure, and anthropometry data were then assessed. This included measurements of body height, mass, and skinfold thickness at four sites (biceps, triceps, subscapular, and supra-iliac) to estimate body fat (Durnin and Womersley, 1974). Subsequently, aerobic fitness was assessed using a submaximal cycle ergometry test (Finger et al., 2013). Exercise testing was performed in the upright position with an electronically braked cycle ergometer (Ergometrics 900, Ergoline, Bitz, Germany). After a resting period of 5 min, where the subjects were instructed to sit quietly and relax on the cycle ergometer, the incremental bicycle protocol started with the subject pedaling at 25 W for 2 min. The power output was then increased by 25 W every 2 min until the subject reached a target heart rate of 150 bpm. We encouraged all subjects to aim to maintain a pedaling frequency of 70 rpm throughout the whole test session. Heart rate was continuously recorded using a HR monitor (RS800CX, Polar Electro, Kempele, Finland). The degree of effort exerted by the participants at the end of each workload was determined using the standardized Borg rating of perceived exertion (RPE) Scale (Borg, 1982). Capillary blood samples for lactate analysis (Enzymatic-Amperometric Measuring System, Hitado super GL2 analyzer, Dreihausen, Germany) were taken prior to starting the test as well as at the end of each workload stage.

Special software (ERGONIZER, Freiburg, Germany) was used for the investigator-independent calculation and was based on an equalizing SPLINE interpolation procedure. The lactate threshold (LT) determined from this interpolated curve over the minimum of the quotient lactate/power output was read as the start of increase in lactate concentration (Roecker et al., 1998).

To describe aerobic fitness, we determined two submaximal indicators of endurance performance:

1. Physical work capacity during heart rate of 150 bpm, which represents the power output at a heart rate of 150 bpm, and was determined using a heart rate-power output plot.
2. Lactate threshold, which represents the first increase in blood lactate concentrations above resting values and demarcates the upper limit of a range of exercise intensities (moderate exercise domain) that can be accomplished almost entirely aerobically.

The determination of these two parameters offers the following great advantages compared to parameters such as maximum oxygen uptake (VO_2_max): (1) maximal effort and motivation are not mandatory during the examination and (2) the testing procedure is less risky.

#### Paradigm to Assess the Placebo Effect

**Figure 3:**
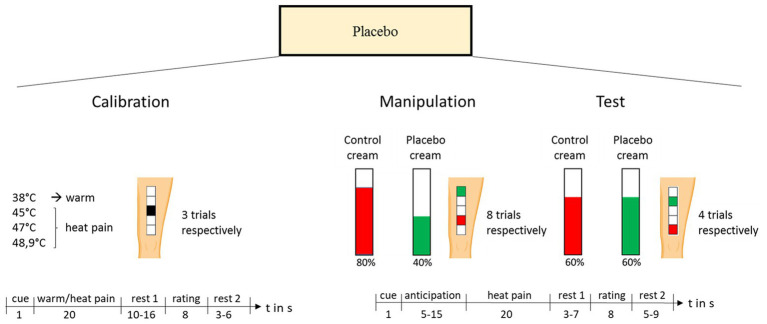
Placebo paradigm. The placebo paradigm consisted of three phases: calibration, manipulation, and test. Before the experiment started, five 4 × 4 cm^2^ squares were drawn on the participants’ left thigh to mark the stimulation sites. Subjects were informed that they would receive pain-inhibition cream/inactive control cream on the skin areas outlined in green/red. The two upper/lower squares were outlined in green/red and designated as the site for later placebo/control cream stimulation. The assignment of placebo cream or control cream to the upper/lower patches was randomized across subjects. During the calibration phase, the middle square was used to determine the individual temperatures to evoke pain levels of 40, 60, and 80 on the VAS, ranging from 0 = no pain to 100 = unbearable pain. Therefore, a pseudo-random sequence of 12 20-s thermal stimuli with different intensities (38, 45, 47, and 48.9°C, three trials, respectively) was applied while the participants were asked to rate the intensity of each stimulus on the presented VAS. The individual temperatures evoking VAS ratings of 40, 60, and 80 were calculated *via* linear regression of the calibration ratings. Before the manipulation phase started, participants were told that they would be stimulated with the same pain level on both skin patches (placebo cream and control cream). Unbeknown to them, they were stimulated with a pain level of VAS 80 to the control site and with a pain level of VAS 40 for stimuli applied to the placebo site. The conditioning phase consisted of a pseudo-random sequence of placebo cream/control cream stimulation with eight trials each. The test phase consisted of a pseudo-random sequence with four trials per stimulation site. Importantly, participants were stimulated with the same temperature (equivalent to 60 on the VAS) at both stimulation sites (placebo-control). This physically identical stimulation allowed for the assessment of the individual placebo analgesic effect (i.e., reduced pain ratings under placebo cream compared with control cream).

On visit 2, a well-established placebo paradigm (Wager et al., 2004; Eippert et al., 2009; Stein et al., 2012), including both expectation and conditioning components (Amanzio and Benedetti, 1999), was conducted. The paradigm consisted of three phases: calibration, manipulation, and test. The experimental design for the placebo test is shown in Figure 3. Before the experiment started, five 4 × 4 cm^2^ squares were drawn on the subjects’ left thigh to mark the stimulation sites. Then, two identical-looking, pharmacologically inactive creams were applied, which were introduced as analgesic cream and control cream and were kept in professionally labeled tubes. The participants were informed that they would receive pain-inhibition cream/inactive control cream on the skin areas outlined in green/red. The two upper/lower squares were outlined in green/red and designated as the site for later placebo cream/control cream stimulation. During the calibration phase, the middle square was used to determine the individual temperatures to evoke pain levels of 40, 60, and 80 on the visual analogue scale (VAS) ranging from 0 = no pain to 100 = unbearable pain. Therefore, a pseudo-random sequence of twelve 20-s thermal stimuli with different intensities (38, 45, 47, 48.9°C, three trials, respectively, baseline temperature = 34°C, approximately 1.5-s ramp up, 17-s plateau, approximately 1.5-s ramp down) was applied using a 27-mm-diameter fMRI-compatible Peltier thermode [PATHWAY model, contact heat-evoked potential stimulator (CHEPS); Ramat Yishai, Israel]. Participants were asked to rate the intensity of each stimulus on the presented VAS. The individual temperatures evoking VAS ratings of 40, 60, and 80 were calculated *via* linear regression of the calibration ratings. Before the manipulation phase started, participants were told that they would be stimulated with the same pain level on both skin patches (placebo cream and control cream). Unbeknown to them, they were stimulated with a pain level of VAS 80 to the control site and with a pain level of VAS 40 for stimuli applied to the placebo site. The conditioning phase consisted of a pseudo-random sequence (stimulation of skin patch treated with control/placebo cream) with eight trials each. Each trial consisted of pain anticipation, noxious stimulation, rest 1, pain rating, and rest 2. During the anticipation phase, a red/green crosshair appeared on the screen announcing the stimulation at the control/placebo site. After a variable delay (5–15 s), a 20-s thermal stimulus was administered (baseline temperature = 34°C, approximately 1.5-s ramp up, 17-s plateau, approximately 1.5-s ramp down). After another short delay (3–7 s), participants had to rate the level of pain on the VAS. Then, a variable intertrial interval followed. The test phase consisted of a pseudo-random sequence with four trials per stimulation site. Importantly, participants were stimulated with the same temperature (equivalent to 60 on the VAS) at both stimulation sites (placebo-control). This physically identical stimulation allowed for the assessment of the individual placebo analgesic effect (i.e., reduced pain ratings under placebo cream compared with control cream). The experimental design for the placebo test is shown in Figure 3.

#### Paradigm to Assess Conditioned Pain Modulation

**Figure 4:**
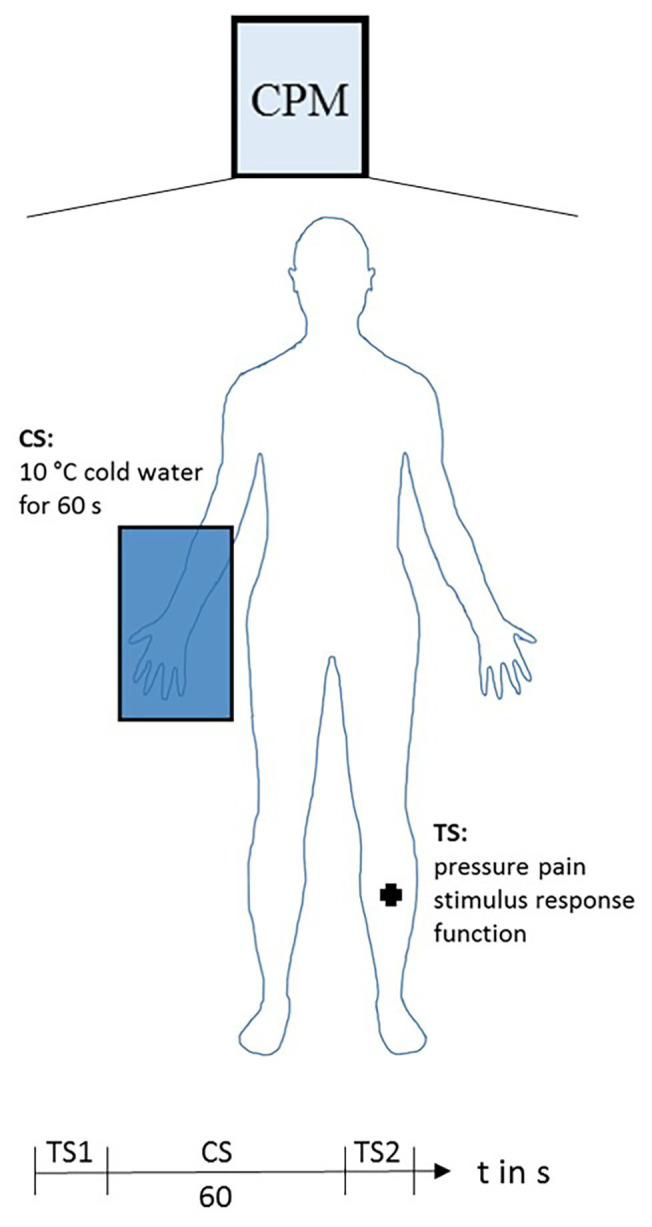
Conditioned pain modulation paradigm. Test stimulus (TS): stimulus-response function of painful pressure at left leg. Conditioned stimulus (CS): 10°C cold water immersion of the right hand for 1 min. Pain was rated after 45 s of the CS on a VAS ranging from 0 = no pain to 100 = unbearable pain. Sequence of consecutive applied stimuli: first test stimulus (TS1), CS, second test stimulus (TS2).

After the placebo experiment, the classical CPM paradigm (Yarnitsky et al., 2015) was performed (see Figure 4). A stimulus-response function of painful pressure was used as the TS (Geisler et al., 2019b). The pressure stimuli (probe area of 1 cm^2^) were applied to the tibia bone of the left leg with a constantly increasing intensity of 50 kPa/s using an algometer deep tissue transducer and a computer-based VAS response unit (SENSEBox, Somedic AB, Hörby, Sweden). As a reference point, the midpoint of the distance between the distal end of the patella and the medial malleolus was marked at the skin. Subjects scored the sensations continuously using the computer-based 100-mm VAS unit with the left end (0 mm) indicating “no pain” and the right end (100 mm) “maximal pain.” Subjects were instructed to say “stop” when the maximal bearable pain was reached. The stimulation was immediately stopped when the tolerance threshold or, to avoid any tissue damage, when a maximum stimulation intensity of 1,500 kPa was reached (Geisler et al., 2019a). A computer sampled the stimulus intensity at a rate of 100 Hz and the corresponding VAS rating. After the first test stimulus (TS1), the conditioned stimulus (CS) was applied. Therefore, subjects had to immerse their right hand up to the wrist in 10°C cold water for 1 min. After 45 s, the CS subjects had to rate the pain on a VAS ranging from 0 = no pain to 100 = unbearable pain. The second test stimulus (TS2) was applied immediately after the CS. To prevent/reduce sensitization or habituation, the stimulation place between TS1 and TS2 was slightly changed. We defined the CPM effect as the difference of VAS_TS2_ − VAS_TS1_ during a fixed pressure (*z*-standardized pressure = 0, see “Data Analysis” section for details). Thus, a negative value indicates a decrease in VAS rating at TS2 and, therefore, CPM-induced pain inhibition.

#### Questionnaires

We assessed several psychological variables, which have been shown to modulate pain perception, CPM (Geva and Defrin, 2013; Geva et al., 2017), or placebo (Enck et al., 2013) to control for differences between groups: depression [Beck depression-inventory-II (BDI II), Beck et al., n.d.], positive and negative affect [Positive and Negative Affect Schedule (PANAS); Watson et al., 1988; Krohne et al., 1996], state and trait anxiety [state and trait anxiety inventory (STAI-G); Laux et al., 1981], optimism [Life-Orientation-Test-Revised (LOT-R); Glaesmer et al., 2008], extraversion, neuroticism, psychoticism, and social desirability [short version of Eysenck Personality Questionnaire-Revised (EPQ-RK); Ruch, 1999], pain catastrophizing [Pain Catastrophizing Scale (PCS); Meyer et al., 2008], interoceptive awareness [Multidimensional Assessment of Interoceptive Awareness (MAIA); Mehling et al., 2012], extraversion, agreeableness, conscientiousness, emotional stability, openness [Big-Five-Inventory-10 (BFI-10); Rammstedt et al., 2013], and pain anxiety [Pain Anxiety Symptoms Scale (Pass-20-GV); Kreddig et al., 2015]. The German version of the questionnaires was used. All participants filled out the questionnaires assessing psychological variables at home between study visits.

### Data Analysis

All data were analyzed using R version 3.4.1 (R Core Team, 2017). We used the serial gatekeeping approach to test our H1–H4. Thus, we continued the testing of the hypothesis only when the previous hypothesis had been accepted; otherwise, the procedure would stop (Turk et al., 2008). Significance levels were set to *p* ≤ 0.05.

#### Demographic Data

We compared demographic data (biographical data, results of questionnaires and fitness characteristics) between groups using independent sample *t*-tests when data were normally distributed and using the Mann–Whitney *U* test otherwise.

#### Conditioned Pain Modulation

As some participants (4/17 nonathletes and 11/16 athletes) did reach the maximum stimulation intensity before reaching the tolerance threshold during TS1 or TS2, we first checked the data of all participants visually. This quality control revealed a strong linear relationship between the VAS rating and the applied pressure in all participants. We used lmerTest (Kuznetsova et al., 2017) to perform a hierarchical linear modeling (HLM) to analyze CPM. We decided to analyze the data using a multilevel approach for several reasons: first, our data has a clear multilevel structure; in fact, the observation from the stimulus-response function of painful pressure (level 1) is nested within subjects (level 2). Thus, the multilevel analysis allows the simultaneous examination of the effects of predictors on a subjects’ level (group comparison) and on a stimulus-response function level. Second, multilevel analysis accounts for the nonindependence of observations within subjects (Diez-Roux, 2000). Third, multilevel analysis allows testing multiple hypotheses (in the current study, H1 and H2) within a single test, minimizing the alpha error cumulation. Last, using this approach, we were able to calculate the best-fitting stimulus-response function as a linear regression for each subject at level 1, respectively, even if there were some missing values.

In the specified model, the observation from the stimulus-response function of painful pressure (level 1) was nested within subjects (level 2). Thus, level 1 describes within-subject variance, whereas level 2 captures between-subject variation. The model includes the maximal random effect structure justified by the data (Barr et al., 2013), respectively. At level 1, a simple linear regression with random intercept and random slope was modeled for the stimulus-response function of painful pressure with *VAS* rating as the dependent variable and *z*-standardized *Pressure* as the independent variable [see Equations (1) and (4)]. We *z*-standardized (subtracting the mean of all data points from each individual data point, then dividing those points by the standard deviation of all points) *Pressure* because the initial model using *Pressure* failed to converge due to a very large eigenvalue ratio. At level 2, we modeled a multiple linear regression for the intercept *β*_0*j*_ and the slope *β*_1*j*_ of level 1, respectively, with the indicator variables *CPM* (TS2 = 0, TS1 = 1), *Group* (nonathletes = 0, athletes = 1), and the interaction term *CPM Group* as predictors [see Equations (2) and (3)]:

CPM level 1:

(1) $$VAS_{ij}=\beta_{0j}+\beta_{1j}•Pressure+\varepsilon_{ij}$$

CPM level 2:

(2) $$\begin{array}{l} (\beta_{0j}=\gamma_{00}+\gamma_{01}•CPM+\gamma_{02}•Group\\ \;\;\;\;\;\;\;\;\;\;\;\;\;\;\;\;\;\;\;+\gamma_{03}•CPM•Group+u_{00}+u_{01}•CPM \end{array}$$

(3) $$\begin{array}{l} \beta_{1j}=\gamma_{10}+\gamma_{11}•CPM+\gamma_{12}•Group+\\ \;\;\;\;\;\;\;\;\;\;\;\;\;\;\;\gamma_{13}•CPM•Group+u_{10}+u_{11}•CPM \end{array}$$

CPM total equation:

(4) $$\begin{array}{l} VAS_{ij}=\gamma_{00}+\gamma_{01}•CPM+\gamma_{02}•Group+\\ \;\;\;\;\;\;\;\;\;\;\;\;\;\;\;\;\;\;\;\;\;\gamma_{03}•CPM•Group+\\ \;\;\;\;\;\;\;\;\;\;\;\;\;\;\;\;\;\;\;\;\left(\begin{array}{l} \gamma_{10}+\gamma_{11}•CPM+\gamma_{12}•Group+\\ \gamma_{13}•CPM•Group \end{array}\right)\\ \;\;\;\;\;\;\;\;\;\;\;\;\;\;\;\;\;\;\;\;•Pressure+u_{00}+u_{01}•CPM+\\ \;\;\;\;\;\;\;\;\;\;\;\;\;\;\;\;\;\;\;\left(u_{10}+u_{11}•CPM\right)\\ \;\;\;\;\;\;\;\;\;\;\;\;\;\;\;\;\;\;\;•Pressure+\varepsilon_{ij} \end{array}$$

We define the CPM effect as the difference VAS_TS2_ − VAS_TS1_ during *z*-standardized pressure = 0. Thus, a negative value indicates a decrease in the VAS rating at TS2 and, therefore, CPM-induced pain inhibition (Yarnitsky et al., 2015).

#### Placebo Effect

We define the individual placebo effect as PE_i_ = ∆VAS_i_∙$\frac{40}{\Delta Mi}$,where ∆VAS_i_ is the mean difference between VAS ratings of the control and placebo sites during the test phase, 40 is the expected mean difference between VAS ratings of the control and placebo sites during the manipulation phase, and ∆M_i_ is the observed mean difference between VAS ratings of the control and placebo sites during the manipulation phase (Stein et al., 2012). Thus, the individual placebo effect is the absolute placebo effect ∆VAS_i_ but controlled for individual differences of conditioning strength during the manipulation phase. A positive value of PE_i_ indicates a decrease in VAS rating at the placebo site compared to the control site during the test phase (in which stimulation intensities were the same for both sites) and, therefore, placebo-induced hypoalgesia. We compared PE_i_ between groups using an independent sample *t*-test.

#### Association Analysis

To test H4, we calculated a correlation between CPM and placebo. Furthermore, we exploratively analyzed the associations between CPM and PWC150, CPM and LT, placebo and PWC150, and placebo and LT. Last, we exploratively analyzed the associations between demographic variables, which differ significantly between groups (see Supplementary Table S1) and placebo/CPM, respectively. These include correlation analyses between placebo/CPM and HR variability (RMSSD, SDNN), interoceptive awareness (MAIA_total, MAIA_noticing), conscientiousness (BFI_ 10_conscientiousness), and openness (BFI_10_openness). Pearson correlations were calculated when data were normally distributed, and Spearman correlations were calculated otherwise. As the stated explorative analyses are thought to provide potentially additional worthwhile information that could serve to generate hypotheses for future studies but are not directly related to the primary aim of the current study, they do not require any correction for multiplicity (Turk et al., 2008).

## Results

All results in the text are reported as mean ± SD.

### Demographic Data

In accordance with our selection criterion and shown in Supplementary Table S1, our high-endurance athletes and nonathletes differ in all measured parameters of endurance capacity (lower HR at rest, higher PWC150, and higher LT, all *p*s < 0.001). Furthermore, athletes had higher HR variability [higher root mean sum of squared distance (RMSSD) and SD of the interbeat interval of normal sinus beats (SDNN) during rest; all *p*s < 0.001], confirming enhanced vagal modulation through chronic endurance exercise training (Sandercock et al., 2005). Analysis of personality questionnaires reveals only significant group differences in interoceptive awareness (MAIA_total_athletes_ > MAIA_total_nonathletes_ and MAIA_noticing_athletes_ > MAIA_noticing_nonathletes_), conscientiousness (BFI_ 10_conscientiousness_athletes_ > BFI_10_ conscientiousness_nonathletes_), and openness (BFI_10_openness_athletes_ < BFI_10_openness_nonathletes_; all *p*s < 0.05; See Supplementary Table S1).

### Conditioned Pain Modulation

Nonathletes rated the pain intensity of CS on average as 83.53 ± 16.75 and athletes as 65.00 ± 30.28. The mean pressure of test stimulation was 760 kPa (equals whole group *z*-standardized pressure of 0). At TS1, nonathletes rated this stimulation intensity as 62.81 ± 28.24 and at TS2 as 62.01 ± 32.32. This reduction of pain rating (−0.80 ± 20.32) equals a mean CPM effect of −1.3%. In contrast, athletes rated the same stimulation intensity of 762 kPa at TS1 as 43.05 ± 33.18 and at TS2 as 26.64 ± 25.34, which results in a mean CPM effect of −38.1%.

We performed a multilevel analysis to evaluate H1 [athletes rate pressure pain as less intense (*γ*_02_ < 0) and have a lower slope of stimulus-response curve (*γ*_12_ < 0)] and H2 [greater CPM-induced pain inhibition in athletes vs. nonathletes (*γ*_03_ < 0)] with the stimulus-response function of painful pressure (level 1) nested within subjects (level 2) according to Equation 4.

**Figure 5:**
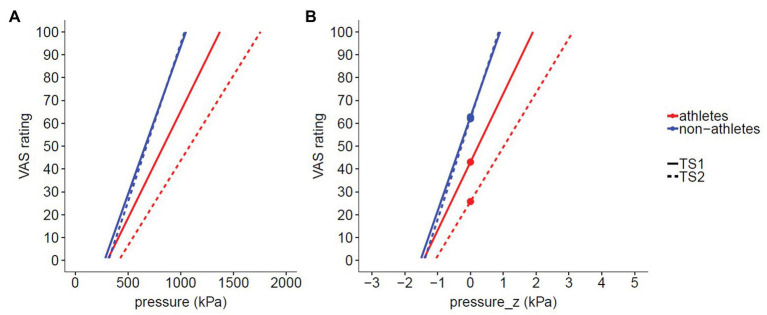
Stimulus response functions (SRF) of pressure pain. **(A)** Original data of SRF of pressure pain applied immediately before (TS1) and after (TS2) 1 min cold water immersion. **(B)** For multilevel data analysis, we used *z*-standardized pressure (for details, see description in “Materials and Methods” section). The CPM effect was defined as difference VAS_TS2_ − VAS_TS1_ during a *z*-standardized pressure of 0 (thick marked points). Thus, a negative value represents CPM-induced hypoalgesia.

**Figure 6:**
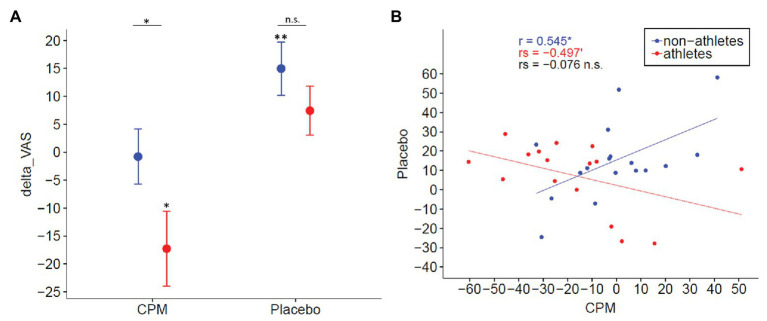
Dissociation of CPM and placebo. **(A)** Mean and standard error of CPM and placebo effects in nonathletes (blue) and endurance athletes (red). A negative value represents CPM-induced hypoalgesia, whereas a positive value represents placebo-induced hypoalgesia. The CPM effect was greater [*t*(32) = −1.983, *p* = 0.023, one-tailed] in athletes than in nonathletes. In contrast, a significant placebo effect was only observed in nonathletes [*t*(15) = 3.148, *p* = 0.006], but not in athletes [*t*(14) = 1.690, *p* = 0.111]. **(B)** Association between CPM effect and placebo effect. Whereas a positive association between CPM and placebo effects [*r*(15) = 0.545, *p* = 0.02] was present in nonathletes, a negative trend was observed in athletes [*r*s(14) = −0.497, *p* = 0.052], indicating that CPM and placebo might be constituted by, at least partially, different mechanisms [*r*s(31) = −0.076, *p* = 0.672]. VAS, visual analog scale.

H1: Athletes rated pressure pain as less intense (*γ*_02_ = −19.764, *p* = 0.038) than nonathletes and showed a lower slope of stimulus response curve (*γ*_12_ = −11.353, *p* = 0.029) compared to nonathletes. As H1 has been accepted, we continued the testing of H2.
H2: There was a significantly greater CPM effect in athletes vs. nonathletes (*γ*_03_ = −16.4, *p* = 0.023). However, we could not find a significant CPM effect in nonathletes (*γ*_01_ = −0.8, *p* > 0.1; see Figures 5, 6A and Supplementary Table S2).

As H2 has been accepted, we continued the testing of H3.

### Placebo Effect

The temperatures to evoke a pain sensation of VAS 40, 60, and 80 as determined in the calibration phase were 46.16 ± 1.48°C, 47.58 ± 1.43°C, and 49.00 ± 1.54°C with no group differences (all *p*s > 0.1). The observed mean difference between VAS ratings of control and placebo sites during the manipulation phase was ∆M = 39.4 ± 17.2 with no group difference (*p* > 0.1), confirming successful conditioning in both groups. We find a significant [*t*(32) = 3.466, *p* = 0.002] individual placebo effect in the whole sample (PE_i_ = 11.3 ± 18.8) equaling 20% reduction in pain ratings for placebo vs. control in the test phase.

H3: The conducted independent sample *t*-test reveals no group difference of the PE_i_ (*p* > 0.1). However, analyzing the placebo effect separately for both groups, the placebo effect only remained significant in nonathletes [*M* = 14.97, 22%, *t*(16) = 3.148, *p* = 0.006] and not in athletes [*M* = 7.42, 18%, *t*(15) = 1.690, *p* = 0.111; see Figure 6A].

As H3 has been rejected, we stopped the testing of H4 as the primary outcome. Instead, we exploratively analyzed the correlation between the placebo and CPM effects to provide potentially additional worthwhile information that could serve to generate hypotheses for future study.

### Explorative Association Analyses

There is no association between CPM and placebo effects [*r*s(31) = −0.076, *p* = 0.672] when analyzing the whole group. However, a positive association between CPM and placebo effects [*r*(15) = 0.545, *p* = 0.02] is present in nonathletes, and a negative trend is observed in athletes [*r*s(14) = −0.497, *p* = 0.052; see Figure 6B].

**Figure 7:**
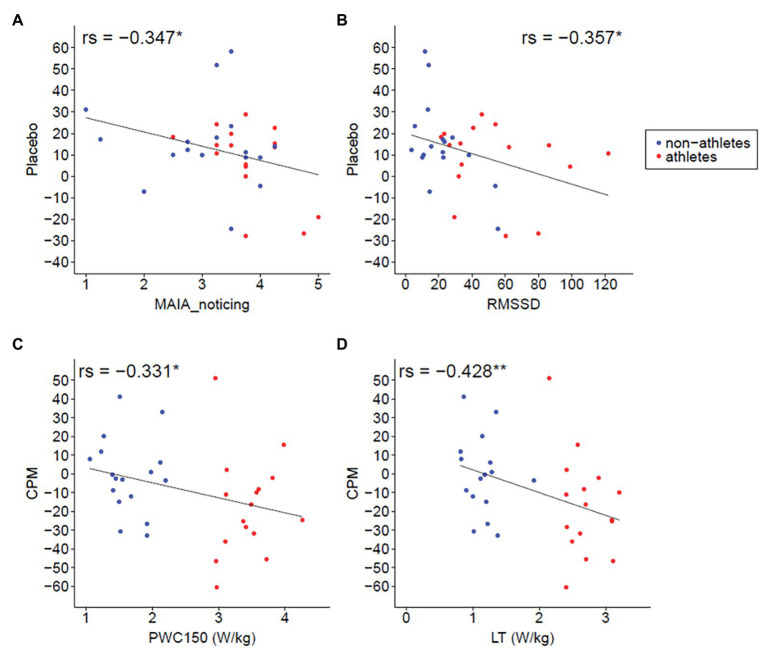
Association analyses. **(A)** Correlation of interoceptive awareness [multidimensional assessment of interoceptive awareness (MAIA)] and placebo. The higher the interoceptive awareness rating, the less strong the placebo effect [*r*s(31) = −0.347, *p* = 0.048]. **(B)** Correlation of HR variability (RMSSD) and placebo. The higher the HR variability, the less strong the placebo effect [*r*s(31) = −0.357, *p* = 0.042]. **(C)** Correlation of PWC150 and CPM. The higher the PWC, the stronger the CPM effect [*r*s(32) = −0.331, *p* = 0.030, one-tailed]. **(D)** Correlation of lactate threshold (LT) and CPM. The higher the LT, the higher the CPM effect [*r*s(31) = −0.428, *p* = 0.008, one-tailed; 1 missing LT value].

Furthermore, we find a negative association between PWC 150 and CPM effect [*r*s(32) = −0.331, *p* = 0.030, one-tailed], as well as between LT and CPM effect [*r*s(31) = −0.428, *p* = 0.008, one-tailed; one missing LT value], indicating a stronger CPM effect in subjects with higher aerobic fitness levels (see Figures 7C,D).

An additional exploratively analyzed correlation reveals no association between placebo/CPM and conscientiousness, placebo/CPM and openness, CPM and interoceptive awareness, CPM and heart rate variability (HRV), placebo and PWC150, and placebo and LT (all *p*s > 0.1). Interestingly, there is a negative association [*r*s(31) = −0.347, *p* = 0.048] between placebo and interoceptive awareness (MAIA_noticing). This means that an increase of interoceptive awareness is associated with a decrease of the placebo effect (Figure 7A). We also find a negative association between placebo and HRV [RMSSD: *r*s(31) = −0.357, *p* = 0.042; SDNN: *r*s(31) = −0.323, *p* = 0.068; Figure 7B]. *Post hoc* analysis reveals that interoceptive awareness and HRV are positively associated [*r*s(31) = 0.405, *p* = 0.020].

## Discussion

The investigation of individual differences in the ability to activate the endogenous pain-inhibition system is important to optimize nondrug pain-management strategies (Abbasi, 2018). Our results suggest a dissociation of the CPM and placebo effects between endurance athletes and nonathletes. A greater CPM effect is found in athletes than nonathletes in the current study. However, a placebo effect was only seen in nonathletes, whereas no placebo effect was seen in athletes. Furthermore, explorative analyses reveal no correlation between the CPM and placebo effect and a negative association between the CPM effect and endurance capacity. Additionally, correlation analyses reveal negative associations between the placebo effect and HR variability as well as between the placebo effect and interoceptive awareness.

### Athletes Perceive Pressure Pain as Less Painful and Show a Greater CPM Effect

As expected (H1), athletes rate suprathreshold pressure pain as less intense and show a lower slope of stimulus-response curve compared to nonathletes. This means that the reduction in pain-intensity ratings of athletes compared to nonathletes gets greater with higher stimulation intensity, which is in line with the results of a meta-analysis by Tesarz et al. (2012). These authors showed that athletes possess higher pain tolerance thresholds than nonathletes, whereas differences of pain thresholds are less consistent across studies. It is suggested that, although no appraisal is needed to detect when a stimulus becomes painful (pain threshold), the rating of suprathreshold stimuli requires appraisal, which mainly depends on previous pain experiences (Geva and Defrin, 2013). Endurance athletes voluntarily proceed with their training routine and/or competition despite arising pain. Thus, their appraisal of pain might change over the years of intense training, or alternatively, only persons with an already changed appraisal of pain become endurance athletes. Furthermore, athletes have a higher CPM effect than nonathletes, confirming H2 and in accordance with recent studies on triathletes (Geva and Defrin, 2013; Geva et al., 2017) and athletes of different kinds of sports, including cycling, swimming, football, weightlifting, and martial arts (Flood et al., 2016). Only one study reports a reduced CPM effect in endurance athletes compared to nonathletes (Tesarz et al., 2013). In that study, the second test stimulus was presented 1 min after the CS, which could have been too late to assess the peak CPM effect as the effect is generally short-lived (Kakigi, 1994; Yarnitsky et al., 2015). Interestingly, there is a lack of CPM effect in our group of nonathletes. We cannot exclude that the higher perceived intensity of the CS in nonathletes (about 20 VAS units) might have influenced the CPM efficiency. Indeed, it is shown that, although the pain intensity of the CS does not influence the magnitude of CPM within the same stimulus modality (Razavi et al., 2014), the perceived CS pain can influence the CPM magnitude (Nir et al., 2012). Nir et al. (2012) showed that cognitively decreasing the perceived CS pain (in a placebo condition) decreases the CPM magnitude. However, as this study reveals no change in the CPM magnitude in a nocebo group in which the CS pain was perceived as more painful, we think that the lack of CPM effect in our group of nonathletes is most likely not the result of an enhanced perception of CS pain. Interestingly, Flood et al. (2016) also described no CPM effect in their group of nonathletes, whereas Geva and Defrin (2013) analyzed subjects participating in amateur physical exercise up to three times a week as control group. Therefore, future studies are required to investigate whether a minimum amount of exercise per week is required to generate a CPM effect.

### CPM Correlates With Fitness Level

By calculating explorative correlation analyses, we find a negative association between the CPM effect (recall that a negative value represents CPM-induced hypoalgesia) and objective measures of cardiorespiratory fitness (PWC150 and LT). An association between CPM effect and cardiorespiratory fitness has not yet been described although this relationship results directly from the idea that endurance athletes increase their aerobic capacity through years of intensive training and therewith improve their ability to endogenously inhibit pain (Geva and Defrin, 2013). Remarkably, additional explorative analysis reveals no other significant correlations of the CPM effect and the examined demographic variables that differed significantly between groups.

### No Greater Placebo Effect in Athletes

Interestingly, our placebo results dissociate from the CPM results. Analyzing our primary study objective (H3), we could find a significant endogenously pain reduction through placebo only in nonathletes, whereas no significant placebo effect was detected in athletes. Nonathletes show a reduction of pain rating of 22% during placebo compared to the control stimulation. This is the same amount of pain reduction as described in a study by Wager et al. (2004) using a similar placebo paradigm. Three other studies using the same or similar placebo paradigms found slightly different placebo-induced pain reduction (13%, Grahl et al., 2018; 28%, Stein et al., 2012; and 36%, Eippert et al., 2009). Surprisingly, the placebo effect in athletes is not significant. This cannot be explained by a loss of the ability to endogenously inhibit pain as athletes show an even stronger CPM pain inhibition effect than nonathletes.

### Association of Placebo Effect and Interoceptive Awareness

In our study, athletes indicate higher interoceptive awareness and HRV than nonathletes, and exploratively, correlation analysis reveals a negative association between the placebo effect and interoceptive awareness as well as between the placebo effect and HRV. It has been shown that nonelite runners use a strategy to dissociate painful input during a run, whereas elite long distance runners often use an associate strategy, which highlights that they listen to their body signals and adjust running pace accordingly (Morgan and Pollock, 1977). Thus, high interoceptive awareness might be a result of such an associate strategy. Moreover, it is possible that persons with high interoceptive awareness also use an associate strategy during placebo stimulation. Research on placebo identifies several psychological, neuroendocrine, and genetic variables that modulate the individual placebo effect (for review, see Enck et al., 2013). However, Buchel et al. (2014) recently proposed a new predictive coding theory of placebo hypoalgesia. They argued that the probability of the perceived pain can be estimated when the probabilities of the sensory input and the prior expectation are given. Thus, the amount of the placebo effect only depends on prior expectations, including the precision of the expectation and the sensory input. Grahl et al. (2018) argued that all modulator variables identified modulate the placebo effect by changing the precision of expectation or the expectation itself. With regard to the idea that subjects with high interoceptive awareness (athletes) use an associate strategy during placebo stimulation, the predictive coding theory predicts that, in athletes, the variance of the probability of the perceived sensory input is smaller (more precise), and therewith, the peak becomes higher than in persons with lower interoceptive awareness (nonathletes). The more precise probability of the perceived sensory input leads to a faster reduction of the prediction error and, therewith, to a smaller placebo effect provided the probability of the treatment expectation is similar between subjects. This interpretation is in line with a recent study showing that accurate pain reporting training diminishes the placebo response (Treister et al., 2018). Furthermore, it is recently shown that HRV and interoceptive awareness are highly positively correlated in healthy subjects (Owens et al., 2018). We also find a positive association between HRV and interoceptive awareness. Thus, the negative correlation of the placebo effect and HRV could be an epiphenomenon of the relation between interoceptive awareness and the placebo effect.

### CPM and Placebo Effects Might Rely on Different Mechanisms

Considering both main results of our study, the greater CPM effect in athletes than in nonathletes and a significant placebo effect in nonathletes but no significant placebo effect in athletes, it is very likely that pain inhibition by placebo and CPM do not rely on the same mechanisms. This hypothesis is further supported by the nonsignificant correlation of CPM and the placebo effect (H4). Moreover, there is a positive association between CPM and the placebo effect in nonathletes, whereas we observe a negative trend in athletes. Our results are in line with a study by Skyt et al. (2018). They find no association between CPM and the placebo effect in neuropathic pain patients. Furthermore, using fMRI, Youssef et al. (2016) explored the brainstem mechanism of CPM in detail and find that, among others, the PAG is not critical for CPM-induced hypoalgesia in humans, confirming results from animal research (Bouhassira et al., 1990). Instead, they suggest a spino-bulbo-spinal loop with critical involvement of the subnucleus reticularis dorsalis (SRD). Thus, CPM possibly involves other key structures in the brainstem (e.g., SRD) and, therewith, other top-down pain inhibition pathways and involved neurotransmitters than placebo (Damien et al., 2018).

### Limitations and Future Directions

The present study has several limitations. First, we use different stimulation modalities (heat pain vs. pressure pain) to evaluate the placebo and CPM effects. It is shown that the individual placebo effect depends on type of pain (Liberman, 1964). Therefore, future studies should investigate whether the dissociation between the placebo and CPM effects remains when the same stimulation type is used for both paradigms. Second, we only analyzed men. Thus, our drawn conclusion should not be generalized to women. Future studies should also include female populations. Third, the colors used in the placebo paradigm and the sequence of both paradigms (placebo followed by CPM) have not been counterbalanced. Therefore, we can exclude neither different effects of colors on pain perception nor that the placebo paradigm might have biased the results of the CPM paradigm. However, we think that these effects should not have an influence on our reported group difference results as both groups (athletes and nonathletes) underwent exactly the same experimental conditions, and therewith, any effects by chance should have been averaged out. In particular, we see no plausible reason why male athletes should differentially react to the colors as compared to male nonathletes. Fourth, our study design included a rest period of about 30 min between the placebo and CPM paradigms. However, we have to acknowledge that this delay might not be sufficient to exclude any cross-contamination. It is possible that exhaustion of the pain inhibitory system due to the placebo paradigm might have influenced the results of the CPM paradigm. However, as the primary aim of our study was to test whether athletes have an increased placebo effect and as we first assessed the placebo effect, these results are unaffected by any other test. Furthermore, we could find a greater CPM effect in athletes compared to nonathletes and, therewith, replicated a number of recent studies on CPM (Geva and Defrin, 2013; Flood et al., 2016; Geva et al., 2017). Therefore, we think that the influence of the repeated testing of pain inhibition on the CPM results, if present, should be low. Future studies should realize different examinations on different days to be sure that paradigms do not influence each other. Fifth, we did not blind the experimenter as there were obvious signs that allowed the experimenter to guess whether a subject is an athlete (strong muscles, very low heart frequency during rest, etc.) or not. However, as the instructions were the same for participants from both groups and as all participants were naïve to the hypotheses of the study, we think that the results of our study are reliable.

Sixth, we did not include a 60-s non-CPM control condition in our paradigm and, therewith, cannot exclude that the larger CPM effect in athletes is partly driven by a larger habituation. However, to minimize any habituation effects, we used a slightly different area for TS2 application (counterbalanced over subjects). Seventh, due to the relatively small sample size, we have to acknowledge that our result that there is no group difference in placebo effect might be a Type 2 error. However, as athletes have a numerical lower placebo effect than nonathletes, we are quite confident that it is unlikely that they have truly a *greater* placebo effect than nonathletes (as hypothesized).

Eighth, the described association between placebo effect and interoceptive awareness and HRV come from explorative analyses. Future studies are highly recommended to replicate these interesting relations.

In summary, our study reveals a dissociation for CPM and placebo effects between athletes and nonathletes and, therewith, provides evidence that these two effects that represent the ability of endogenous pain control are based, at least in part, on different biological mechanisms.

## Data Availability Statement

The raw data supporting the conclusions of this article will be made available by the authors, without undue reservation.

## Ethics Statement

The studies involving human participants were reviewed and approved by The Ethics committee of the Faculty of Social and Behavioural Sciences of the Friedrich Schiller University Jena. The patients/participants provided their written informed consent to participate in this study.

## Author Contributions

MG, K-JB, and TW designed the experiment. MG and MH collected and analyzed data. All authors discussed the results, revised the article critically for important intellectual content, and approved the final article.

### Conflict of Interest

The authors declare that the research was conducted in the absence of any commercial or financial relationships that could be construed as a potential conflict of interest.
