# Supplementary material for: Dissociation of Endogenous Pain Inhibition Due to Conditioned Pain Modulation and Placebo in Male Athletes Versus Nonathletes
Source: Front Psychol. 2020 Sep 18;11:553530. doi: 10.3389/fpsyg.2020.553530 (PMC7531190; doi:10.3389/fpsyg.2020.553530)
Supplement: Supplementary file 1 [file Data_Sheet_1.docx]

**Supplemental information**

**Table S1. Demographic characteristics of subjects**

|  | **athletes** | | **non-athletes** | |  |
| --- | --- | --- | --- | --- | --- |
|  | ***M*** | ***SD*** | ***M*** | ***SD*** | ***p*** |
| *biographical data*  age (years)  BMI (kg/m²)  endurance sport (h/week)  *physical fitness*  PWC150 (W/kg)  LT (W/kg)  *physio data PWC-test rest*  HR  RMSSD  SDNN  *heat pain ratings (VAS 0-100)*  38°C  45°C  47°C  48.9°C  *personality questionnaires*  BDI II  STAI_G_X1  STAI_G_X2  MAIA_total  MAIA_noticing  MAIA_not_distracting  MAIA_not_worrying  MAIA_attention_regulation  MAIA_emotional_awareness  MAIA_self_regulation  MAIA_body_listening  MAIA_trusting  PANAS_positiv  PANAS_negativ  Pain_Catastrophyzing  BFI_10_extraversion  BFI_10_agreeableness  BFI_10_ conscientiousness  BFI_emotional_stability  BFI_10_openness  EPQ_RK_extraversion  EPQ_RK_neuroticism  EPQ_RK_psychoticism  EPQ_RK_social_desirability  LOT_R_pessimism  LOT_R_optimism  PASS_20_GV_total | 27.9  22.9  9.3  3.4  2.67  65.36  53.03  78.13  2.6  26.9  46.1  72.7  2.6  36.4  33.3  25.0  3.8  2.0  2.3  3.4  3.6  3.1  2.4  4.4  3.9  1.9  13.3  3.4  3.3  3.8  2.3  3.2  7.3  2.6  2.1  3.4  3.3  9.4  28.3 | 5.0  1.6  3.9  0.4  0.31  9.07  29.86  24.17  3.4  14.6  17.1  17.0  2.7  7.3  7.4  3.2  0.6  0.8  0.6  0.6  0.7  0.9  1.0  0.5  0.4  0.5  7.6  1.0  0.8  0.7  0.8  1.1  3.9  2.6  1.3  2.6  1.5  1.8  16.2 | 26.9  24.1  0  1.6  1.15  92.67  21.49  39.83  4.6  29.7  52.8  76.3  3.7  33.4  36.3  21.6  3.1  1.9  2.4  2.9  3.0  2.5  1.8  3.9  3.5  2.0  17.2  3.2  3.2  3.3  2.7  4.0  7.2  3.8  2.1  2.8  3.9  8.7  33.7 | 6.3  3.2  0  0.4  0.27  10.77  15.12  20.09  7.3  20.0  20.3  14.8  4.5  7.2  9.7  4.1  0.9  0.6  0.6  0.8  1.25  1.0  1.3  0.9  0.7  0.7  9.6  1.1  0.8  0.8  0.8  1.0  3.1  3.6  1.4  1.6  2.3  2.7  15.9 | 0.621  0.193  **< 0.001^a^**  **< 0.001**  **< 0.001**  **<0.001^a^**  **<0.001^a^**  **<0.001**  0.650^a^  0.957^a^  0.314  0.525  0.756^a^  0.250  0.470^a^  **0.013**  **0.014**  0.855^a^  0.446  0.063  0.142  0.118  0.113  0.153^a^  0.116  0.828  0.211  0.523^a^  0.941^a^  **0.048**  0.204^a^  **0.024^a^**  0.952  0.489^a^  0.955^a^  0.659  0.533^a^  0.545^a^  0.342 |

Group specific mean (*M*) and standard deviation (*SD*) of demographic variables. *P*-value are given for group comparisons (N_athletes_ = 16, N_non-athletes_ = 17) using independent two sample t-tests (df = 31, except for LT df =30 due to one missing value) when data were normally distributed and Mann-Whitney U test otherwise (a). PWC150 = physical work capacity during a heart rate of 150, LT = lactate threshold, HR = heart rate, RMSSD = root mean sum of squared distance, SDNN = standard deviation of the inter beat interval of normal sinus beats, BDI II = Beck depression-inventory-II, PANAS = Positive and Negative Affect Schedule, STAI-G = state and trait anxiety inventory, LOT-R = Life-Orientation-Test-Revised, EPQ-RK = short version of Eysenck Personality Questionnaire-Revised, PCS = Pain Catastrophizing Scale, MAIA = Multidimensional Assessment of Interoceptive Awareness, BFI-10 = Big-Five-Inventory-10, Pass-20-GV = Pain Anxiety Symptoms Scale.

**Table S2.** Results of multilevel analysis for conditioned pain modulation (CPM)

| **Fixed effects** | | | | | **Random effects** | |
| --- | --- | --- | --- | --- | --- | --- |
| **Parameter** | ***β*** | ***SE(β)*** | ***t-value*** | ***p*** | **Parameter** | ***SD*** |
| *γ_00_* | 62.819 | 7.458 | 8.423 | <0.001 | *u_00_* | 30.74 |
| *γ_01_* | -0.834 | 5.761 | -0.145 | 0.443’ | *u_01_* | 23.71 |
| *γ_02_* | -19.764 | 10.710 | -1.845 | 0.038’ | *u_10_* | 16.57 |
| *γ_03_* | -16.408 | 8.272 | -1.983 | 0.023’ | *u_11_* | 16.16 |
| *γ_10_* | -41.250 | 4.026 | 10.247 | <0.001 | *ε_ij_* | 5.68 |
| *γ_11_* | 2.820 | 3.934 | -0.717 | 0.717 |  |  |
| *γ_12_* | -11.353 | 5.780 | -1.964 | 0.029’ |  |  |
| *γ_13_* | -8.928 | 5.647 | -1.581 | 0.124 |  |  |

Estimated values of fixed effect parameters (*β*), corresponding standard errors *SE(β)* and p-values (*p*) for t-test of *β* against 0 (‘one-tailed due to directed hypothesis, two-tailed otherwise). Estimated standard deviation (*SD*) of random effect parameters. CPM total equation: *VAS_ij_ = γ_00_ + γ_01_ ∙ CPM + γ_02_ ∙ Group + γ_03_ ∙ CPM ∙ Group + (γ_10_ + γ_11_ ∙ CPM + γ_12_ ∙ Group + γ_13_ ∙ CPM ∙ Group) ∙ Pressure + u_00_ + u_01_ ∙ CPM + (u_10_ + u_11_ ∙ CPM) ∙ Pressure + ε_ij_*
